# *Boule* Is Present in Fish and Bisexually Expressed in Adult and Embryonic Germ Cells of Medaka

**DOI:** 10.1371/journal.pone.0006097

**Published:** 2009-06-30

**Authors:** Hongyan Xu, Zhendong Li, Mingyou Li, Li Wang, Yunhan Hong

**Affiliations:** Department of Biological Sciences, National University of Singapore, Singapore, Singapore; Duke University, United States of America

## Abstract

**Background:**

The *DAZ* family genes *boule*, *daz* and *dazl* encode RNA binding proteins essential for fertility of diverse animals including human. *dazl* has bisexual expression in both mitotic and meiotic germ cells, whereas *daz* has male premeiotic expression, and *boule* is largely a unisexual meiotic regulator. Although *boule* has been proposed as the ancestor for *dazl/daz* by gene duplication, it has been identified only in invertebrates and mammals. It has, however, remained unclear when and how the DAZ family has evolved in vertebrates.

**Methodology and Principal Findings:**

This study was aimed at identifying and characterizing the *DAZ* family genes in fish as the basal vertebrate. We show that *boule* and *dazl* coexist in medaka and stickleback. Similar to the medaka *dazl* (*Odazl*), the medaka *boule* (*Obol*) is maternally supplied and segregates with primordial germ cells. Surprisingly, *Obol is* expressed in adult germ cells at pre-meiotic and meiotic stages of spermatogenesis and oogenesis. However, the maximal meiotic *Obol* expression in spermatocytes contrasts with the predominant pre-meiotic *Odazl* expression in spermatogonia, and the diffuse cytoplasmic *Obol* distribution in early oocytes contrasts with the *Odazl* concentration in the Balbinani's body.

**Conclusions:**

The identification of fish *boule* and *dazl* genes provides direct evidence for the early gene duplication during vertebrate evolution. Our finding that *Obol* exhibits bisexual expression in both embryonic and adult germ cells considerably extends the diversity of *boule* expression patterns and offers a new insight into the evolutions of DAZ family members, expression patterns and functions in animal fertility.

## Introduction

Germline development and gametogenesis proceed in multiple processes that are complex and apparently divergent among metazoans. Understanding of the mechanisms underlying these processes is crucial for fertility and reproductive medicine in human as well as for germline engineering in animals. The *DAZ* (*Deleted in Azoospermia*) gene family offers one of the few lines of evidence that argue for evolutionary conservation of these processes at the molecular level. The *DAZ* family comprises *daz*, *dazl* and *boule*. DAZ family proteins are characterized by a conserved ribonucleoprotein (RNP)-type RNA recognition motif (RRM) and one (Boule and Dazl) or multiple repeats (Daz) of the DAZ motif. Historically, the DAZ family was established with the identification of the founder member *Daz*. In human, multiple copies of Daz genes cluster on the Y chromosome [1], [2]. Deletion of the Daz cluster is associated with azoospermia and oligospermia, making Daz a strong candidate for the Azoospermia factor. In addition, the human has an autosomal *Daz*-like gene, termed *Dazla* [3], [4]. The Y-chromosomal Daz is limited to catarrhine primates, whereas the autosomal Dazl homologue has been found in mouse [5]. Accordingly, the autosomal *Dazla* has been proposed to be the ancestor of the *Daz* cluster, with its duplication followed by transposition to the Y chromosome having occurred during the evolution of primates [2]. Dazl homologues have been identified also in all major groups of non-mammalian vertebrates including chicken [6], Xenopus [7], axolotl [8], zebrafish [9], medaka [10] and gibel carp [11]. The third family member *boule* was found in *Drosophila* as a gene essential for the germline development [12]. Similarly, in the nematode *Caenorhabditis elegans*, a gene named as *daz*-1 was identified as the boule/dazl counterpart [13]. In all organisms examined, the *DAZ* family genes are exclusively expressed in, and required for, the germline development. The notion that *daz, dazl* and *boule* are evolutionary homologues is supported by observation that the human *Dazl* can rescue the *Drosophila boule* mutant to some extent [14]. Because vertebrates have both *boule* and *dazl*, and invertebrates merely have *boule*, it has been proposed that *boule* is the ancestor of the *DAZ* gene family. During the vertebrate evolution, *boule* underwent gene duplication, producing the autosomal *dazl*, which duplicated further, leading to additional copies that were translocated to the Y-chromosome in catarrhine primates and amplified to multiple *daz* genes [15]. Indeed, *boule* has recently been described in few mammals including human [15], mouse [15], sheep [16] and cattle [17].

In spite of germline expression, different *DAZ* family genes have considerable diversity in sex- and stage-specific expression. Human *Daz* and *Drosophila boule* are transcribed specifically in the male germline [1], [2], [18]. Human *Dazla* and its mouse, zebrafish, and *Xenopus* homologues are expressed in the germline of both sexes [7], [9], [19], [20], [21]. In the nematode *daz-1* (*boule/dazl* homologue) mRNA is expressed in germ cells mostly in female [22], albeit its protein is detectable in germ cells in male as well [23]. In sheep, *boule* is also highly expressed in both male and female gonads undergoing meiosis as analyzed by RT-PCR [16].

The role of the *DAZ* family genes in the production of gametes is still largely unknown, partly due to the apparent diversity of phenotypes caused by their defects. A human male missing the DAZ cluster shows a varying range of defects in spermatogenesis, from no germ cells at all to less severe spermatogenic arrest generating some mature spermatids [1], indicating that the DAZ cluster is not absolutely necessary for the entry into meiosis and sperm production. Targeted disruption of mouse *Dazla* results in the complete absence of gamete production in both sexes, with *Dazla*-defective mice having female germ cells arrested at prophase of meiosis I and male germ cells being affected at the proliferating stage [19]. In contrast, the *Drosophila boule* is essential for the meiotic progression in spermatogenesis but not oogenesis, as mutant flies have male germ cells arresting at the G2/M transition in meiosis I, exhibiting limited postmeiotic differentiation [12]. In the nematode, mutations of the *boule* homologue *daz-1* completely abolishes fertility in hermaphrodites due to arrest at meiotic prophase in oogenesis, while *daz-1*-defective males exhibit no significant defect and are fully fertile [13], [22], [23]. Thus, although the *DAZ* family genes encode regulators of gametogenesis, their sex specificity varies considerably depending on the family member and species.

With the identification of human and mouse *boule* in addition to *dazl* and *daz*, Xu et al [15] proposed a model for the *DAZ* gene family evolution. According to this model, *boule* was the ancestor gene that underwent the first gene duplication, generating autosomal *dazl*; *dazl* during primate evolution underwent the second gene duplication and chromosomal translocation to the Y chromosome, resulting in *Daz*; and two more *Daz* gene duplication produced the present-day *Daz* clusters in human. There are two puzzles in understanding of the DAZ family evolution. First, *boule* usually has unisexual meiotic expression in both invertebtates and mammals, whereas *dazl* has bisexual expression in both premeiotic and meiotic germ cells, raising an interesting question as to whether the first vertebrate *boule* homologue has unisexual or bisexual germline expression. The second puzzle is concerned with the timing of the first *boule* gene duplication leading to *dazl*. If this gene duplication indeed occurred early in vertebrate evolution as hypothesized [15], all vertebrate species must have both *boule* and *dazl*. However, *boule* in vertebrates has so far been identified only in mammals, raising the question as to whether *boule* is restricted to mammals by lineage-specific gene duplication. Since the separation between the fish and tetrapod lineages ultimately leading to mammals occurred at the beginning of vertebrate evolution approximately 450 million years ago, fish represent an ideal system to explore the DAZ family evolution in the basal vertebrates.

Previously, we have identified *dazl* in the fish medaka and revealed its expression in embryonic and adult germ cells of both sexes [10]. This study was aimed at the identification of *boule* in medaka (*Oryzias latipes*), and both *boule* and *dazl* in a second fish species, namely the three spined stickleback (*Gasteroteus aculeatus*). Furthermore, we examined the medaka *boule* RNA expression in developing embryos and adult gonads. Medaka is an excellent model for investigating germline development and gametogenesis *in vivo* and *in vitro*. It has a completely sequenced genome facilitating gene identification and reverse genetics, male germ stem cell line capable of sperm production *in vitro* [24], the ease of observation of germline development and robust genetic manipulations in embryos [25] and stem cell cultures [26]. We showed that the medaka *boule* has a previously unidentified expression pattern, which may provide important implications on the *DAZ* gene family evolution and *boule* gene function.

## Results and Discussion

### Fish Possess both *boule* and *dazl*

**Figure 1:**
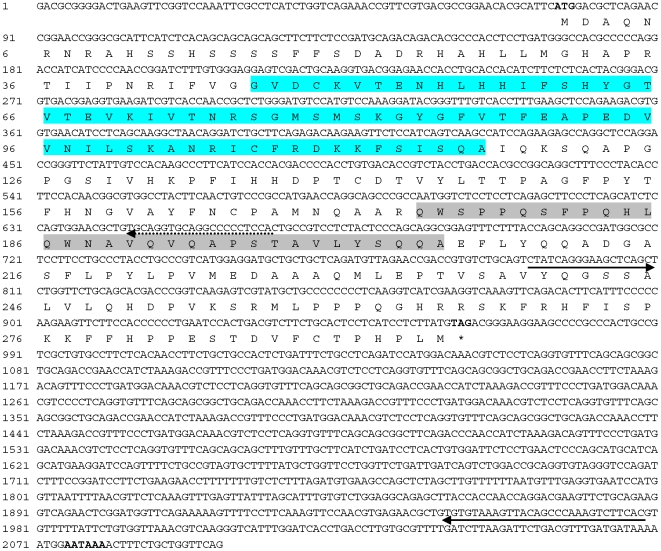
Nucleotide sequence of the medaka *boule* cDNA. O*bol* and its deduced protein OBol: shown in bold are the translation start codon, stop codon and putative poly-adenylation signal. Highlighted are RRM motif (Turquoise) and DAZ motif (light grey). Underlined are primer sequences for 5′ RACE (dash) and RT-PCR (solid, fragment for RNA probe) with arrows depicting their extension directions.

**Figure 2:**
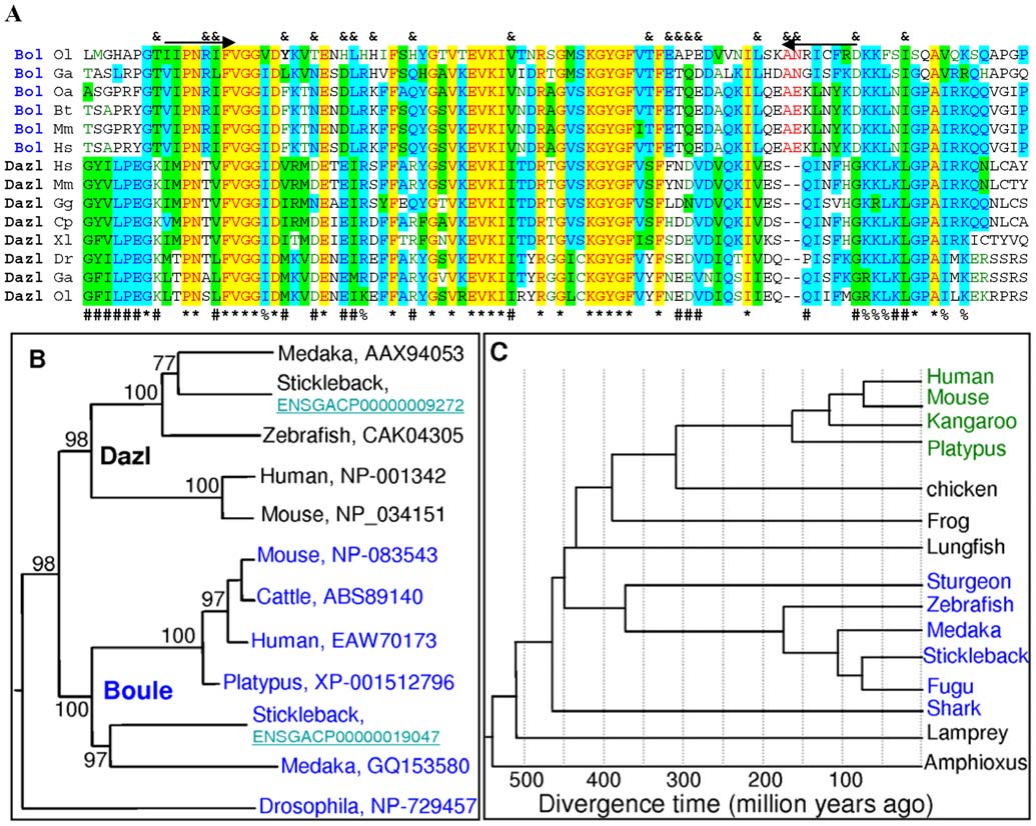
Identification of fish *boule* and its expression in medaka. (A) Multiple sequence alignment of the RRM. Boule and Dazl proteins share 27 invariant residues (asterisks) and seven conserved positions (%). There are 20 invariant or conserved residues characteristic of Boule (&) or 21 of Dazl (#) proteins each. Ol, *Oryzias latipes* (medaka); Ga, *Gasterosteus aculeatus* (stickleback); Gg, *Gallus gallus* (chicken); Oa, Ornithorhynchus anatinus (platypus); Bt, *Bos taurus* (cattle); Mm, *Mus musculus* (mouse); Xl, *Xenopus laevis* (African clawed frog); Cp, *Cynops pyrrhogaster* (newt); Hs, Homo sapiens (human). (B) Phylogenetic tree of DAZ family proteins. Notably, the branching between Boule and Dazl clades coincides with the branching between the fish and tetrapod lineages, and molecular trees on the basis of either Boule or Dazl sequences are well in accordance with organism relationships, indicating that generation of boule and Dazl took place in early vertebrate evolution. Followed species are gene accession numbers. (C) The phenogenic relationship of major bony fish with other vertebrates and their divergence time: Bony fish groups in blue; Mammal groups in green and other transit groups in dark; drawings are based on references [27], [42] and data from http://en.wikipedia.org/wiki/Timeline_of_human_evolution#Primates.

The DAZ family proteins show apparent sequence variations in diverse organisms except for the RRM and DAZ motif. To determine whether *boule* was present in the fish lineage, we exploited molecular cloning and bioinformatics approach and chose the medaka as the first model to identify *boule* and *dazl* homologues in fish. Since *Odazl*, the medaka *dazl*, has previously been cloned [10], here we focused on cloning *boule* gene in the strain i^3^ medaka. We cloned the *boule* gene before the medaka genome sequence became available. A multiple sequence alignment of invertebrate and mammalian Boule proteins led to the identification of two conserved sequences for degenerate primers (see below). RT-PCR with the degenerate primers resulted in a partial sequence that was found to be most similar to the human BOULE. Rapid amplification of cDNA ends (RACEs) by using gene-specific primers led to the composition of a full length cDNA that was cloned by using terminal primers and verified by sequencing. This cDNA appears to be the medaka *boule*, termed *Obol*. *Obol* is 2,097 nt in length, contains a 74-nt 5′ untranslated region (UTR), a 1,134-nt 3′ UTR and a 885-nt open reading frame (ORF) encoding a protein of 295 amino acid residues (aa) (Fig. 1). The predicted protein OBol possesses a conserved RRM and a DAZ repeat (Fig. 1). OBol is 41% and 42% identical in sequence to the mouse and human BOULE, respectively, and 28% to the human DAZL (data not shown), indicating it is more similar to Boule than to Dazl. The conserved positions usually reside within the RRM (Fig. 2A).

The cloning of *Obol* in this study and *Odazl* in our previous work enabled us to search for *boule* and *dazl* homologues in other fish species. Blast search against sequenced fish genomes (www.ensembl.org) resulted in the identification of single *boule* (ENSGACP00000019047) and *dazl* (ENSGACP00000009272) genes also in stickleback (*Gasterosteus aculeatus*). A multiple sequence alignment of the RRM reveals that Boule differ from Dazl proteins in 20 invariant and/or conserved positions, besides 27 invariant and/or conserved positions common to both Boule and Dazl (Fig. 2A). On a phylogenetic tree, fish and mammalian Boule proteins are clustered together, whereas all Dazl forms a separate clade (Fig. 2B). As illustrated in Fig. 2C, the stickleback belongs to the Percomorpha, separating about 100 million years ago from the Beloniformes to which the medaka belongs. Together, Percomorpha and Beloniformes are within the lineage Acanthopterygii and separated approximately 180 million years ago from the Ostariophysii to which the zebrafish belongs[27]. Interestingly, the branching into the Boule and Dazl clades coincides with separation between fish and tetrapod lineages. This, together with the fact that fish Boule is more similar to mammalian Boule than to fish Dazl, strongly suggests the early appearance of vertebrate *boule* before fish-tetrapod separation about 450 million years ago.

**Figure 3:**
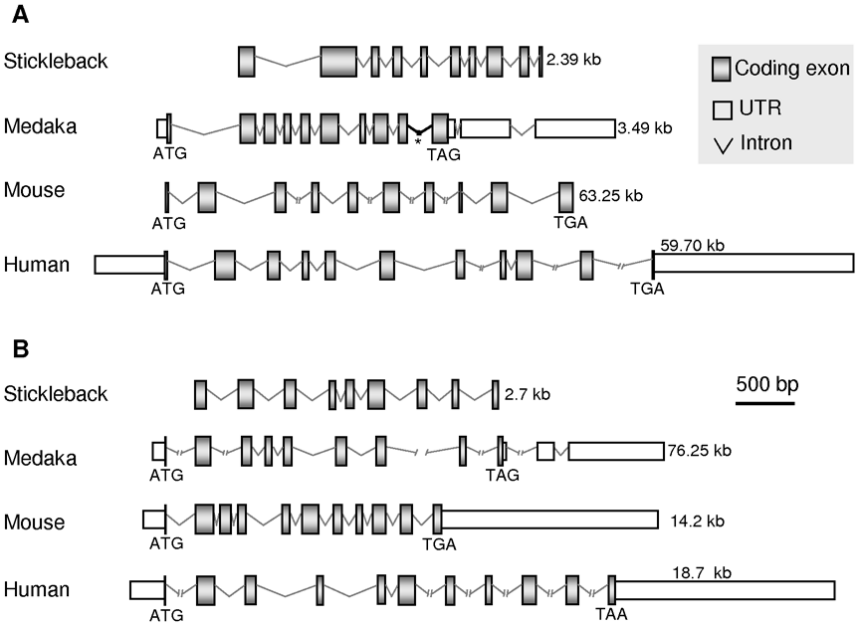
Comparisons of genomic organizations between fish and mammal. A. *boule*. B.*dazl*. Exons are shown in scale. The sizes of primary transcripts are indicated. The 5′ and 3′ untranslated regions (UTR) are not known for the stickleback *boule* and *dazl*. The fish genes are generally smaller than the human genes except the medaka *dazl* that is the largest. Notably, both medaka *boule* and *dazl* have introns in the region coding for 3′-UTRs. There is a gap (*) in the intron between the medaka *boule* exons.

**Figure 4:**
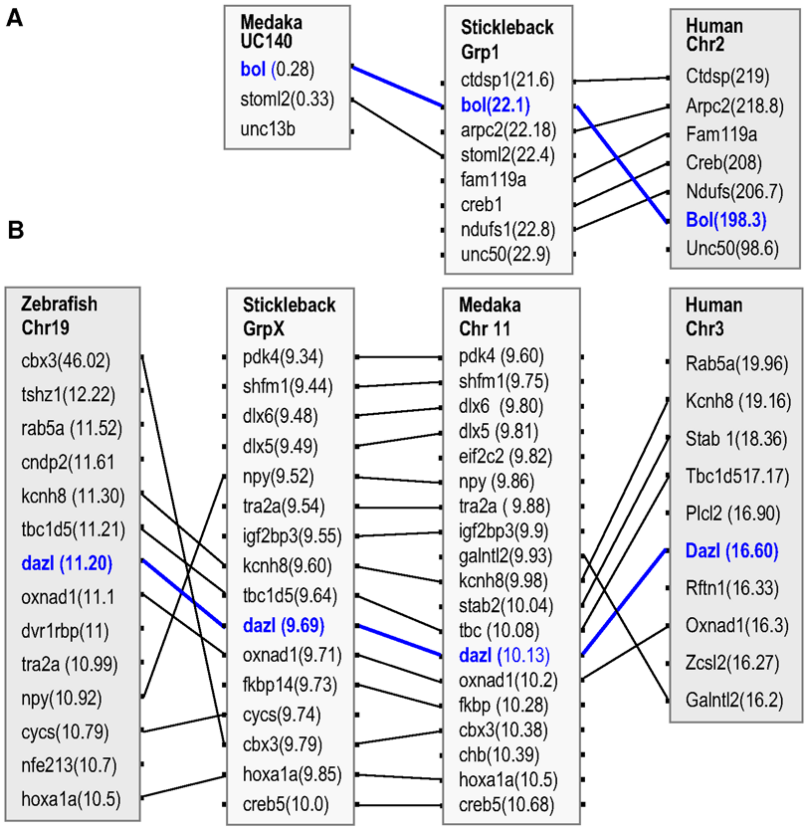
Chromosome synteny of fish *boule* and *dazl* genes. (A) Chromosomal synteny of *boule* gene. (B) Chromosomal synteny of *dazl* gene. Chr, chromosome; grp, linkage group; UC, ultracontig. Numericals in parentheses are chromosomal positions.

Fish *boule* and *dazl* resemble their mammalian counterparts in gene structure consisting of 12 and 11 exons, respectively (Fig. 3). To further determine whether fish *boule* and *dazl* were the evolutionary orthologues of human Boule and Dazl respectively, we analyzed the chromosomal syntenic relationships. *Obol* is adjacent to *stoml2* gene on Ultracontig 140 that is syntenic to a small region of stickleback chromosome/group 1. This region contains eight genes including *boule* and is fully syntenic to the Boule-bearing region on human chromosome 2 (Fig. 4A). On the other hand, the *dazl*-bearing regions on different fish chromosomes (medaka Chr11, stickleback group X and zebrafish Chr19) exhibit a conserved synteny to the *Dazl*-containing human chromosome 3 (Fig. 4B). Taken together, fish have both *boule* and *dazl* genes, demonstrating that the gene duplication leading to *boule* and *dazl* must have taken place before the separation between the fish and tetrapod lineages. Furthermore, the *DAZ* gene family in fish very probably has only two members, namely *boule* and *dazl*, because blast searches by using *boule* and *dazl* queries failed to detect any additional *DAZ* family genes in fish cDNA databases and all of the five sequenced genomes (data not shown). This situation is reminiscent of non-primate mammals and indicates that either *boule* or *dazl* has undergone no or rare additional gene duplication after fish-tetrapod separation. In accordance with this notion is the presence of a putative *boule* also in chicken (XP_421917), in addition to a described chicken *dazl* [6]

### *Boule* has a Novel Expression Pattern in Medaka

**Figure 5:**
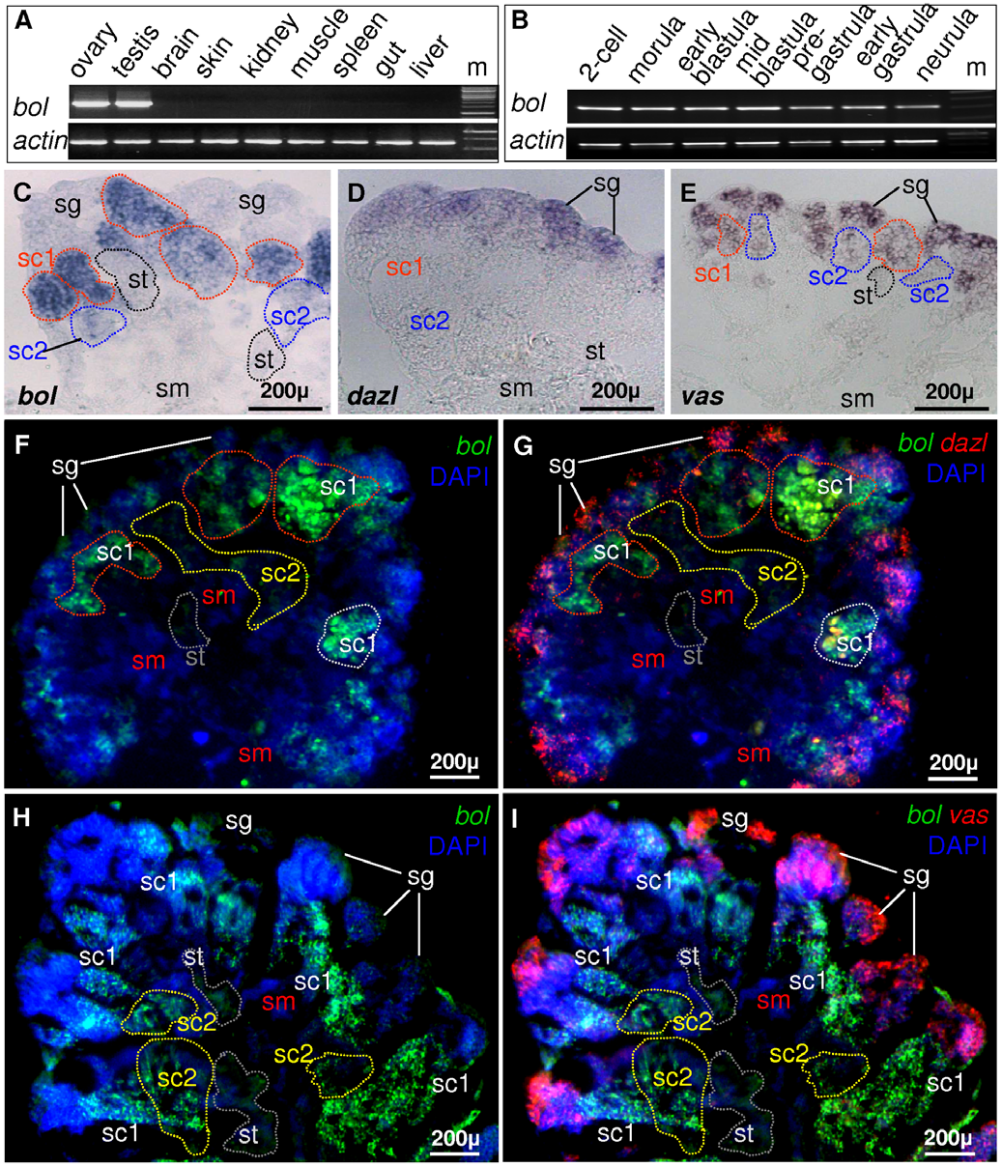
Embryonic, adult and spermatogenic RNA expression. (A-B) Adult (A) and embryonic expression (B) by RT-PCR. (C-I) Spermatogenic expression by in situ hybridization. Adult testicular cryosections were hybridized to antisense RNA probes and the signals were visualized by chemical (C-E) and fluorescent staining (F-I). Nuclei were stained with DAPI (blue). (C-E) Chemical SISH. (C) *Obol* probe. (D) *Odazl* probe. (E) *Olvas* probe. (F-I) Dual color FISH. (F and G) *Obol* and *Odazl* FISH. (H and I) *Obol* and *Olvas* FISH. *Obol*, *Odazl* and *Olvas* show distinct stage-preferential expression patterns. The *Obol* signal peaks in primary spermatocytes and reduces in secondary spermatocytes and spermatids. Notably, spermatogonia exhibit a low and detectable *Obol* signal. In contrast, the *Odazl* signal peaks in spermatogonia and sharply reduces in spermatocytes, whereas the *Olvas* signal peaks in spermatogonia and persists at reduced levels from spermatocytes to spermatids. sg, spermatogonium; sc 1 and sc2, primary and secondary spermatocyte; st, spermatid; sm, sperm.

In all species examined so far, *dazl* has bisexual germline expression, while *boule* usually has unisexual expression. Moreover, *boule* also shows sex specificity in different organisms, namely male fly [12] and mammals [15], but female worm [22]. RT-PCR analyses revealed that the adult *Obol* RNA expression is absent in somatic tissues but high in adult gonads of both sexes (Fig. 5A), suggesting that adult *Obol* expression may be restricted to germ cells. Amazingly, the *Obol* RNA is easily detectable in early developing embryos (Fig. 5B) before the midblastula stage when bulk zygotic gene transcription takes place [28], demonstrating that *Obol* RNA is maternally supplied.

In both fly and mammals, *boule* expression occurs in male meiotic germ cells, and its deficiency causes male sterility by meiotic arrest in *Drosophila*. We performed section in situ hybridization (SISH) to examine *Obol* expression during spermatogenesis and compared with *Odazl* and *Olvas*, the well-studied germ cell markers in diverse animals [8], [10], [29], [30], [31]. The adult medaka testis is composed of seminiferous lobules, each comprising cysts of germ cells synchronously at various stages of spermatogenesis. Cysts of spermatogonia are located at the most peripheral region. Spermatogenesis proceeds synchronously within each cyst, and cysts of germ cells at progressively advanced stages of development reside closer to the efferent duct, which is in the central region. This allows unambiguous definition of individual spermatogenesis stages. SISH by using an antisense RNA probe revealed that the O*bol* transcript was most abundant in meiotic cells, namely spermatocytes. A faint signal was observed also in the meiotic products spermatids. Interestingly, spermatogonia at the testicular periphery also exhibit a weak but detectable signal (Fig. 5C). In contrast, the *Odazl* signal peaks in spermatogonia and reduces in spermatocytes and disappears in post meiotic stages (Fig. 5D). On the other hand, *Olvas* displays an expression pattern overlapping with, but distinctive from, *Obol* and *Odazl* (Fig. 5E). Sense probes did not detect reproducible staining (data not shown). To precisely compare the spatial expression pattern between *Obol* and *Odazl* or *Olvas*, we performed sensitive fluorescent *in situ* hybridization (FISH). As shown in Fig. 5F-I, *Obol* is clearly detectable in certain spermatogonia, and its maximal meiotic expression in spermatocytes alternates with the preferential premeiotic expression of *Odazl* and *Olvas* in spermatogoina. These results demonstrate that *Obol* owns a distinct expression pattern from *Odazl* and *Olvas*, and its expression occurs in both premeiotic and meiotic stages of medaka spermatogenesis.

**Figure 6:**
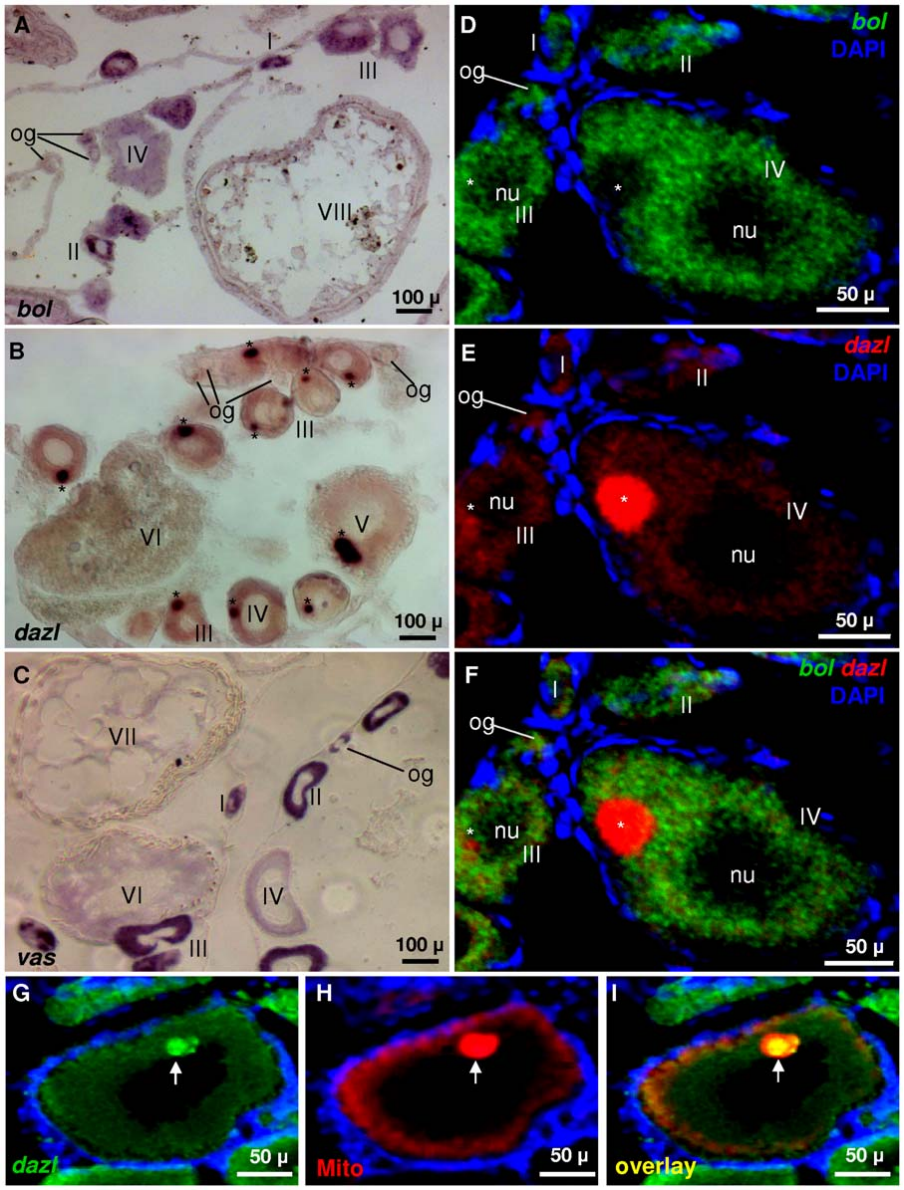
Oogenic RNA expression. Adult ovarian cryosections were hybridized to antisense RNA probes and the signals were visualized by chemical (A-C) and fluorescent staining (D-I). Nuclei were stained with DAPI (blue). (A-C) Chemical SISH. (A) *Obol* probe. (B) *Odazl* probe. (C) *Olvas* probe. (D-F) Dual color FISH. (D) *Obol* signal (E) *Odazl* signal. (F) Merge of *Obol* and *Odazl* signals. The transcripts of *Obol*, *Odazl* and *Olvas* all are not detectable in somatic cells, and their germline expression persists throughout oogenenesis that proceeds in 10 stages (I – X). However, there are distinct differences. Notably, *Obol* and *Odazl* exhibit detectable expression in oogonia, albeit at a lower level than *Olvas*. (G-I) Colocalization of the *Odazl* RNA with mitochondrial cloud in the Balbiani's body of oocytes. (G) *Odazl* probe. (H) MitoTracker Red580 staining for mitochondrial cloud, the characteristic component of BB (arrows). (I) Merge of *Odazl* signal and MitoTracker staining. Intriguingly, *Odazl* concentrates in the Balbinani's body (asterisks), whereas *Obol* is diffuse in the ooplasm and essentially absent in Balbinani's body. Both *Obol* and *Odazl* are absent in the nuclei (nu). It is noteworthy that fluorescent dyes DAPI and propidium iodide do stain nuclei of oogonia but do not stain nuclei of oocytes in medaka, a similar situation has been described also in zebrafish [43].

We then examined *Obol* expression in the ovary. In medaka, the adult ovary comprises a small number of oogonia and oocytes, and oogenesis proceeds in 10 stages [32]. By chemical SISH, *Obol* expression persists throughout oogenesis, in both premeiotic and meiotic stages (Fig. 6A), with the strongest signal being found in early meiotic cells, namely stage II-V oocytes, and a weak but detectable signal being seen in oogonia. This stage-preferential *Obol* expression is similar to *Odazl* (Fig. 6B) and *Olvas* (Fig. 6C), except that *Olvas* also has high expression in oogonia.

A salient difference in RNA localization between *Obol* and *Odazl* was found in stage III-V oocytes. In these oocytes, *Obol* is diffuse in the ooplasm (Fig. 6A and D), whereas *Odazl* predominantly concentrates in a cytoplasmic structure, the so-called Balbiani's body (BB; Fig. 6B, D-F). Diffuse distribution was seen also for *Olvas* (Fig. 6C) and medaka *dead end* [33]. Double color co-localization by FISH reinforced and further strengthened this distinction between *Obol* and *Odazl* in subcellular localization into the BB. Specifically, *Obol* is undetectable in the BB but abundant in the remaining ooplasm, whereas *Odazl* is highly abundant in the BB but rare in the remaining ooplasm. In addition, premeiotic expression in oogonia is clearly detectable for both *Obol* and *Odazl*. Taken together, *Obol* resembles *Odazl* and *Olvas* in premeiotic and meiotic expression during oogenesis, but differs from *Odazl* in the absence of distribution into the BB.

The BB is a membraneless spherical structure in close contact with the nuclei of early developing oocytes [32], [34] and contains mitochondria, endoplasmic reticulum and several germ plasm components including the *dazl* RNA during early oogenesis in *Xenopus* and zebrafish [11], [34], [35], [36]. Specifically, the BB is characterized by highly concentrated mitochondia called mitochondrial cloud, and mitochondrial staining by e.g. the MitoTracker reagent represents a standard procedure to validate the BB identity in the early oocytes of several organisms including zebrafish [11], [34], [35], [36]. FISH and mitochondrial staining clearly revealed that the spherical structure with localized *Odazl* RNA was indeed within mitochondrial cloud, namely the BB (Fig. 6G-I).

**Figure 7:**
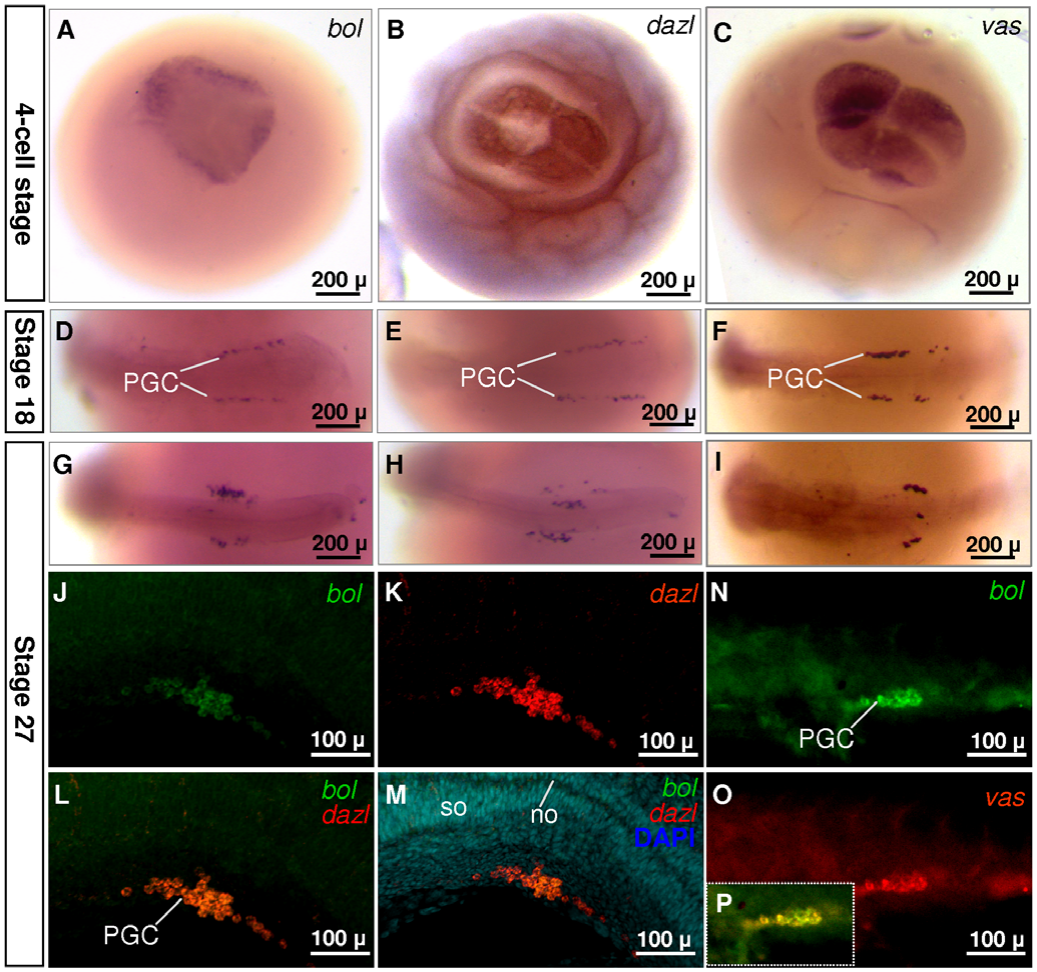
*Obol* is maternally supplied and expressed in PGCs. (A-I) Chemical WISH, showing maternal inheritance (A and C) and PGC expression (D-I) of *Obol*, *Odazl* and *Olvas*. PGCs are seen in two clusters bilateral to the body axis. (J-M) Dual color fluorescent SISH of *Obol* and *Odazl*. (N-P) Dual color fluorescent SISH of *Obol* and *Olvas*. At stage 27, *Obol*, *Odazl* and *Olvas* RNAs colocalize in gondal PGCs in two clusters. so, somites; no, notochord. (A-C) Top view. (D-P) Lateral view. The anterior is to the left.

In Xenopus [7], [21] and fish [9], [10], [37], *dazl* is maternally supplied and expressed in primordial germ cells (PGCs). In contrast, *boule* expression in invertebrates and mammals is restricted to adult germ cells, but absent or unknown in early embryos or PGCs [15]. Our experiments described so far demonstrated that the *Obol* shows an expression pattern distinct from what reported for invertebrate and mammalian *boule*, in that *Obol* has bisexual premeiotic and meiotic expression. These observations, together with early embryonic expression analyzed by RT-PCR (Fig. 5B), provoked us to examine the *Obol* temporospatial expression during embryogenesis. By chemical whole mount *in situ* hybridization, O*bol* was easily detectable in early developing embryos such as the 4-cell stage (Fig. 7A), conforming to its maternal supply as revealed by RT-PCR (Fig. 5B). When embryogenesis proceeds, *Obol* becomes restricted to putative migrating PGCs at stage 18 and post-migratory PGCs at stage 27 (Fig. 7D and G). This *Obol* expression pattern is similar to *Odazl* (Fig. 7B, E and H) [10] and *Olvas* (Fig. 7C, F and I) [29], [38]. To verify that *Obol*-expressing cells were truely PGCs, we performed double color FISH to see whether *Obol-*positive cells were positive also for *Odazl* and *Olvas*. As illustrated in Fig. 7J-P, *Obol* indeed co-localizes with *Odazl* and *Olvas* in the genital PGCs at stage 27. Therefore, we conclude that *Obol* expression occurs in medaka PGCs.

### Conclusions

**Figure 8:**
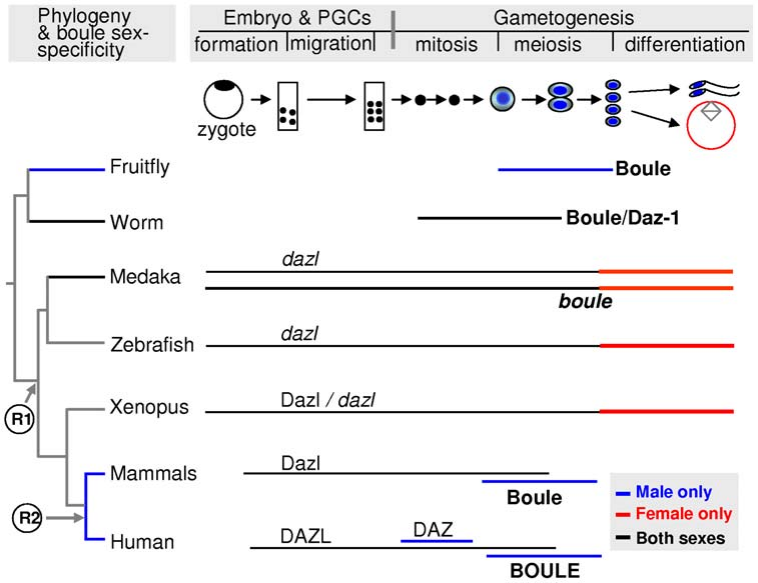
Phylogeny and ontogeny of DAZ family genes. Left, Phylogeny and sex-specificity. The ancient member *boule* exists in all metazoans, whereas *dazl* is in vertebrates and *daz* is restricted to human and certain primates. Evolutionary branching and two gene duplication events (R1 and R2) are indicated. Sex specificity of expression is indicated in different colors. Right, Ontogenic expression. Expression pattern of each member is indicated by extent of horizontal lines. Drawings are originals by author or redrawn based the references [7], [10], [12], [15], [19], [21], [23], [40], [44], [45], [46]. Genes (in italics) refer to RNA expression while Proteins represent the protein expression profiles. Major stages of germline development are diagramed as a timeline for DAZ family gene expression. Expression of *dazl* is detected in many or all stages of germline development in both sexes. Daz has premeiotic male expression. Meiotic expression of Boule occurs in male fly, mouse and human (most abundantly in primary spermatocytes) and mitotic and meiotic in female worm. Data obtained in this study from medaka clearly demonstrate that *boule* is also expressed in embryos at the earliest stage, in primordial germ cells (PGCs) and adult premeiotic and meiotic germ cells of both sexes. The expression of medaka *boule* is similar to that of *dazl* in medaka and other organisms, despite of clear differences as detailed in the text.

In the present study, we report the identification of both *boule* and *dazl* in fish as an ancient vertebrate and the analysis of their expression in medaka embryonic and adult development. Several lines of evidence, including phylogenetic sequence comparisons, protein structure, genomic organization and most convincingly, chromosome synteny, support the notion that the fish *boule* and *dazl* are the orthologues of mammalian *boule* and *dazl*, respectively. With the identification of *boule* in mouse and human, Xu et al. [15] proposed that the *DAZ* gene family has evolved from *boule* by gene duplication and translocation. Our work corroborates and extends this report by demonstrating direct evidence for ancient gene duplication during early vertebrate evolution, prior to the separation between fish and tetrapod lineages approximately 450 million years ago (Fig. 8). The identification of fish *boule* indicates that *boule* and *dazl* coexist widely in the ancient vertebrate. Meanwhile, they become paralogs of each other since the Daz gene family underwent the first duplication.

We have made four striking observations that convincingly demonstrate a totally novel expression pattern for *boule* in medaka. The *boule* RNA expression has been reportedly restricted to meiotic germ cells of male flies and mammals or mainly in female worms but rarely in both sexes, and *boule* deficiency causes sterility by meiotic arrest rather than by abolishing other processes of germline development. Consequently, *boule* is best known as an ancient meiotic gene conserved in metazoans [15]. Here we firstly demonstrate that *boule* in medaka also exhibits a maximal meiotic expression, but this occurs in both sexes, in contrast to its unisexual expression in all other organisms well examined so far. Second, we show that the medaka *boule* also has premeiotic and post-meiotic expression in the adult ovary and testis. Third, we have revealed that medaka *boule* is a maternal message and expressed throughout embryonic germ cell development. Finally, we present evidence that in medaka, *boule* differs from *dazl* in subcellular distribution to the Balbiani's body of early developing oocytes. Taken together, our investigations have revealed a previously unidentified bisexual *boule* expression in embryonic and adult mitotic and meiotic germ cells, further underscoring the expression diversity of DAZ family genes (Fig. 8). Interestingly, *boule* expression in medaka is in a sharp contrast to its unisexual meiotic expression reported in mammals and invertebrates, but is similar to that of medaka *dazl* [10], despite clear differences in preferential expression and stage-specific subcellular localization in the oocytes. The striking finding that *boule* and *dazl* have overlapping but distinct expression patterns provides a new insight into the evolution of *DAZ* family genes and their expressions as well as functions. Since fish separated from tetrapods at the earliest stage of vertebrate evolution, it is tempting to speculate that the medaka *boule* expression may represent the prototype of an ancient vertebrate *boule*. This notion is supported by the identification of putative *boule* and *dazl* genes in a marine fish, and more importantly by similar expression patterns to their medaka counterparts (data not shown). The identification of both *boule* and *dazl* in medaka and stickleback in this study will facilitate the further investigations in other fish species. Further, examination of *boule* and *dazl* expression patterns in other vertebrate species including ancient fishes will help to elaborate the evolution, expression and function of the conserved *DAZ* family genes in (in)fertility of vertebrates including the human.

This study has focused on the RNA expression patterns of both *boule* and *dazl*. It deserved to note that RNA expression pattern may differ from that of the protein products [19], [22], [23], [39], [40]. Therefore, future work is needed to produce antibody against the medaka Boule protein for analyzing its expression and subcellular localization by immunohistochemistry.

## Materials and Methods

### Fish

The medaka was maintained under an artificial photoperiod of 14-h light to 10-h darkness at 26°C. All procedures carried out with medaka fish are conformed to animal care guidelines (Guidelines on the Care and Use of Animals for Scientific Purposes) as outlined by the National Advisory Committee For Laboratory Animal Research in Singapore. Embryogenesis was staged according to Iwamatsu [41]. Oogenesis was according to the 10-stage series of Iwamatsu [32].

### Isolation of cDNA Sequence

Total RNA was isolated from embryos and adult tissues of strain i^3^ medaka by using the Trizol Reagent (Invitrogen). To eliminate genomic DNA contamination, RNA samples were treated with RNase-free DNase (Promega). SMART cDNA libraries were synthesized by using the RACE cDNA Amplification Kit according to the manufacturers' instructions (BD BioSciences). To clone a fragment of the medaka *boule* cDNA, RT-PCR was performed with degenerate primers inferred from conserved amino acid sequences (Fig. 2A). After sequencing, this cloned fragment was used to design gene-specific primers for 5′ and 3′ RACEs as described previously [30]. A full length cDNA sequence was obtained by using terminal primers (ggatccTTCGCGGGCGGGGGAAATGAAGTG and aaaaccgcggACCCTTGACGTTTAAC CACAG; lower case letters, introduced restriction sites for cloning).

### Sequence Analysis

Blast searches were run against public databases by using BLASTN for nucleotide sequences and BLASTP for protein sequences. Multiple sequence alignment was conducted by using the Vector NTI suite 11 (Invitrogen). Phylogenetic tree was constructed by using the DNAMAN package (lynmon Biosoft). Genomic organization and chromosomal locations were investigated by comparing the cDNA and corresponding genomic sequence (http://genome.ucsc.edu/).

### RT-PCR Analysis

The synthesis of cDNA was described [10]. PCR was performed by using two O*bol*-specific primers (bouleF2, aaaactcgagGCAGGCCGATGGCGCCTCC; bouleR2, aaaaccgcggACCC TTGACGTTTAACCACAG). As a control, β-actin was amplified from the same set of cDNA samples using MA1 (TTCAACAGCCCTGCCATGTAC) and MA2 (CCTCCAATCCAG ACAGAGTATT). PCR was run for 30 cycles (*β*-*actin*) 35 cycles (*boule*) of 20 s at 94°C, 20 s at 58°C and 60 s at 72°C. The PCR products were separated on 1.2% agarose gels and documented with a bioimaging system (Synoptics).

### RNA i*n situ* Hybridization

RNA *in situ* hybridization by chemical staining with BCIP/NBT substrates on whole mount samples (WISH) and sections (SISH) were performed as described previously with minor modifications [30]. Briefly, pGEM vectors containing the 1.3 kb partial regions of *Obol* ORF and 3′ UTR (see Fig. 1), 1.8-kb *Olvas* ORF [38] or 984-bp *Odazl* [10] were linearized with Xho I and Sac II and used for the synthesis of sense and anti-sense RNA probes from Sp6 or T7 promoter by using the digoxigenin (DIG) or FITC RNA Labeling Kit (Roche); the RNA probes were treated with RNase-free TURBO DNase and purified (cat# AM1340, Ambion). Multiple color fluorescent in situ hybridization (FISH) was performed by using the TSA™ Plus Fluorescence Systems according to the product manual (NEL756, NEN Life Science). Nuclear staining was done by using 4′–6-Diamidino-2-phenylindole (DAPI) in the Gold Antifade reagent (Invitrogen).

### Mitochondrial Staining

The sections were stained for mitochondria by using the MitoTracker reagent at 100 nM according to the supplier's instruction (M22425, Invitrogen), followed by FISH with the *Odazl* antisense RNA probe as described above and DAPI staining for nuclei before visualization.

### Microscopy and Photography

Observation and photography on Leica MZFIII stereo microscope, Zeiss Axiovertinvert and Axiovert upright microscopes with a Zeiss AxioCam M5Rc digital camera (Zeiss Corp) were as described previously [10]. Confocal images were photographed on Olympus FV500 microscope with the Olympus confocal image system (Olympus). Multiple color fluorescent WISH samples were scanned in a series of 0.5-µm slices and the 3-dimensional images were reconstructed by Z-stacking 150–170 slices on the Huygens (Bitplane AG; Germany).
